# Chronic Trigemino-Cardiac Reflex: An Underestimated Truth

**DOI:** 10.3389/fneur.2017.00022

**Published:** 2017-01-30

**Authors:** Tumul Chowdhury, Bernhard Schaller

**Affiliations:** ^1^Department of Anesthesiology and Perioperative Medicine, University of Manitoba, Winnipeg, MB, Canada; ^2^Department of Research, University of Southampton, Southampton, UK

**Keywords:** trigemino-cardiac reflex, bradycardia, chronic, management, diagnosis

## Abstract

The trigemino-cardiac reflex (TCR) is a brainstem reflex that manifests as adverse cardiorespiratory events upon the stimulation of sensory branches of the fifth cranial nerve. This reflex is mainly investigated in different neurosurgical procedures and intervention. This reflex is commonly considered as an acute and mild physiological response. On the other hand, more devastating and chronic nature of this reflex is largely underreported and unknown. Therefore, this article aims to provide the comprehensive understanding of the chronic form of TCR, its manifestations, and management by literature search. Also, this paper would certainly impart a better diagnosis and understanding of TCR phenomenon by knowing the relatively less common form of a chronic TCR. This will help thousands and thousands of patients who are still in the phase of diagnosis and are suffering from vague symptoms related to this reflex.

## Introduction

The trigemino-cardiac reflex (TCR) is a well-established neurogenic reflex that is mainly investigated in neurosurgical patients (1–6). This unique brainstem reflex is incited by the stimulation of the fifth nerve and produces adverse cardiorespiratory changes including bradycardia, asystole, and apnea. Interestingly, the TCR phenomenon reported in the literature mainly reveals the acute as well as transient nature of this reflex that is considered as a mild physiological response in most of the cases (3, 6). Also, the general management of such situations is the removal of stimuli (3, 6, 7). This relatively high prevalence of detection of the TCR occurrences and the relatively often benign course of the phenomenon is owing to the pre-, peri-, and postoperative presence of the anesthetists (1–9).

However, the chronic nature of TCR event is largely underestimated and underreported in the current literature. This may be partly due to the persistent yet vague nature of symptoms (opposite to the acute TCR episode) that make the diagnosis very difficult. An extrapolation of the prevalence from acute to chronic cases suggests that there might also be a substantial number of chronic cases (10). Therefore, this article aims to provide the comprehensive understanding of the chronic form of TCR, its manifestations, and management by literature search. Also, this paper would certainly impart a better diagnosis and understanding of TCR phenomenon by knowing the relatively less common form of a chronic TCR.

## Method

We searched PubMed for following terms in various combination including (“prolonged” OR “chronic” OR “persistent” OR “delayed”) AND (“oculocardiac reflex” OR “trigeminal cardiac reflex” OR “trigeminocardiac reflex”) from January 1, 1970, to August 31, 2016.

### Definition of Chronic TCR/OCR

Two criteria define chronic TCR. First, TCR/OCR should be explained by the classic definition of this reflex as defined by our previous work (9). The TCR is defined as the sudden onset of parasympathetic dysrhythmia, sympathetic hypotension, apnea, or gastric hypermotility during stimulation of any of the sensory branches of the trigeminal nerve. A TCR should include a decrease in heart rate (HR) and mean arterial blood pressure of more than 20% as compared with baseline values before application of the stimulus and should coincide with the surgical manipulation at or around any branches of the trigeminal nerve (9).

Second, chronicity is defined as any TCR/OCR episodes that persisted at least 1 day before or after the surgery or the first insult.

### Peripheral and Central TCR

OCR and maxilla–mandibular (MCR) TCRs are categorized as peripheral TCR, and any TCR episode due to the stimulation of the fifth nerve pathway beyond the Gasserian ganglion is considered as the central TCR.

### Inclusion

The papers that clearly defined TCR or OCR or MCR as the cause of cardiorespiratory changes were included. All the patients irrespective of age and sex were included. Those papers regardless of the type that has been entirely written in the English language were included. All the relevant cross references were also carefully screened. Pediatric age is defined as the age of less than 18 years.

### Exclusion Criteria

The TCR/OCR episodes that occurred only during the surgical manipulation or within 24 h of surgical stimulation or primary insult were considered as acute TCR and excluded.

## Results

Total of 140 articles were searched through applying the terms. Out of 140, 93 articles met the language and human subject restrictions. Out of 93, only 6 articles finally met the inclusion criteria and further reviewed. Total of six patients included in the final analysis (Table 1). Out of six, 50% were pediatric patients. Strikingly, all the six reported patients were male. Further analysis revealed that the five out of six patients were related to peripheral TCR group and remaining one showed central TCR phenomenon. The timing of presentation of the chronic TCR varied from 29 h to several years. The description of cases is given below.

**Table 1:** **Cases of chronic trigemino-cardiac reflex**.

S no.	Age/gender	injury/procedure	Hemodynamics	Duration	Management	Outcome
1	82/M	MVD of V CN	Bradycardia, cardiac arrest	Several days	Temporary pacemaker	Improved
					Radiofrequency ablation	
2	13/M	Orbital fracture	Bradycardia	29 h	Surgery	Improved
3	12/M	Orbital fracture	Bradycardia	72 h	Surgery	Improved
4	17/M	Foreign body and globe perforation	Bradycardia	48 h	Temporary pacemaker Surgery	Improved
5	Adult/M	Orbital fracture	Bradycardia	1 month	Surgery	Improved
6	56/M	Foreign body in orbit	Bradycardia	40 years	Surgery	Improved

*SN, serial number; MVD of V CN, microvascular decompression of fifth cranial nerve; M, male*.

### The Central TCR Case

This was an octogenarian male patient who underwent a microvascular decompression for a trigeminal neuralgia and developed multiple episodes of bradycardia in postoperative period, and these intermittent adverse cardiovascular changes persisted for several days (11). This patient also developed an event of cardiac arrest and was resuscitated with chest compressions. He was further managed with a temporary pacemaker. Notably, his cardiovascular perturbations coincided with episodes of facial pain. Eventually, he underwent a radiofrequency ablation of fifth nerve and all his cardiovascular and pain related symptoms improved. Strikingly, there were no episodes of such hemodynamics disturbances in the intraoperative period.

### The Peripheral TCR Cases

There were five patients with reported chronic peripheral TCR. In the *first case*, a 13-year-old boy had an orbit medial wall blowout fracture and presented with worsening of nausea and bradycardia 29 h of first insult (12). These symptoms coincided with gazing on rightward direction. An emergency operation was performed, and symptoms got improved after that. In the *second case*, a 12-year-old boy presented with nausea/vomiting and bradycardia (56/min) that intermittently persisted for 72 h after the initial injury (13). He was diagnosed as a case of orbit medial wall blowout fracture. Same day operation was done, and patient’s all symptoms as well as the HR got normalized (68/min). In *the third case*, a 17-year-old male victim of a gunshot at orbit (globe perforation with a foreign body) developed severe bradycardia 48 h after globe perforation repair and persisted for 6 days when a temporary pacemaker was put and eventually foreign body was removed (14). Hemodynamic symptoms reverted to normal. In the *fourth case*, an adult patient who had an orbital floor fracture showed symptoms of nausea, dizziness, bradycardia, sleep disturbances after 1 month of the initial insult (15). He also underwent an operation, and his symptoms got improved. In the *fifth case*, a 56-year-old male war victim presented with features of nausea, vomiting, dyspnea, and bradycardia, 40 years (evisceration was done) after the primary insult (16). At this time, CT scan revealed foreign body in orbit and removed surgically, that completely resolved the symptoms.

## Discussion

The TCR is one of the most investigated brainstem reflexes that manifests commonly as adverse hemodynamic changes during the stimulation of any of the sensory branch of the trigeminal nerve. This phenomenon has been reported in various surgical conditions as well as in neurosurgical interventions and mainly illustrates the acute nature of this reflex that persists during the surgical and other chemical/physical/electrical stimuli (1–10). The afferent nerve fibers carry the stimulus *via* the trigeminal nerve and efferent fibers through the vagus nerve. The TCR is broadly classified as the peripheral and the central type depending upon the stimulation of anatomical location. Depending upon the three sensory divisions of the trigeminal nerve, the peripheral type is further divided into OCR and MCR (9). After reviewing the literature, one can infer that the TCR is a mild transient physiological response and gets abolished after the removal of the inciting stimulus. However, as mentioned in the above-described cases, it can be clearly seen that this phenomenon also has a chronic type and that has far more impact in overall symptomatology and clinical decision-making. We have now gone outside the OR and open the window from a strict cause-effect relationship within a few seconds to a wider variance of reactions (10, 17). Our previous definition work is based on acute cases. We have to look now if these works are also available for the chronic cases. Another point is the connection of chronic TCR to the oxygen-conserving reflex (8). Again, we have no cases of cerebral ischemia undermining a strong correlation between these two phenomena.

Trigemino-cardiac reflex versus vasovagal: the vasovagal response (VVR) can also be postulated as the one of the mechanisms responsible for the similar autonomic manifestations. This reflex mainly controlled through two pathways (18). One is the central VVR that involves cortico-hypothalmic and medullary cardiac centers and second is the peripheral VVR that included heart directly. The VVR is commonly incited by intense emotion, prolonged standing, instrumentation, and severe pain responses (18, 19). This is a transient phenomenon, which improves quickly on lying down, and is usually found in healthy individuals. In described cases, none of the above mentioned inciting factors were present exclusively. In addition, these autonomic manifestations were improved after the surgical interventions, thereby, clearly describes the cause-effect relationship of TCR phenomenon.

There are three significant findings in this review about chronic TCR (11–16). First, the peripheral TCR was the commonest type, and the OCR was the most common subtype associated with the chronic form of TCR. Whether or not the ophthalmic division of the fifth nerve is more prone to the chronic form of TCR is a matter of further investigation. However, considering the present findings of this review, it seems plausible. Also, the first branch of the fifth nerve is also the shortest and pure sensory branch that may be a factor of more incidence of peripheral TCR episodes. Second, besides bradycardia, nausea was the commonest presenting symptom. It is apparent that because of vague symptoms (nausea, vomiting, etc.), the diagnosing the chronic TCR is very challenging. Therefore, it could be another reason for the underreported incidence of chronic TCR cases. Therefore, we want to emphasize here that the TCR should also be included in the differential diagnosis of the patient, if he/she presents with vague symptoms such as nausea, vomiting, dizziness, etc. and had a history of injury (even trivial) including eye, orbit, or any other structure supplied by the fifth nerve. Thirdly, in all the cases, the surgery was the definitive cure for all the symptoms related to the TCR. This important finding suggests that the chronic form of TCR seems more like a pathological rather than a physiological entity, and has a negative impact on the overall health of the patient. Here, it should be noted that the indication of surgery/intervention was mainly due to the on-going symptoms rather than an absolute pathological or surgical cause. Therefore, for the entire medical community, it is imperative to understand the phenomenon of chronic TCR that is not limited to any particular specialty. This will help thousands and thousands of patients who are still in the phase of diagnosis and are suffering from those vague symptoms daily.

## Limitation

In the paucity of substantial evidence, these case reports should only be interpreted as an informative tool for understanding the concept of chronic TCR. These cases do exhibit a sound cause and effect relationship and therefore support our hypothesis. All cases were found in males. It may be a simple reflection of injury pattern that is more common in the male gender (20). Whether or not peripheral TCR is common in males is a matter of further research.

## Conclusion

The chronic form of TCR is an underreported entity and certainly has a substantial impact on the patient health. With the knowledge of the chronic TCR, we can be certainly able to diagnose more and more patients and impart better management in such cases. Further research is warranted to elucidate the exact mechanism of chronic TCR.

## Author Contributions

TC helped in developing the concept, designing, data collection, data interpretation, and writing the manuscript. BS gave substantial inputs in developing the concept and writing the manuscript. Both the authors accepted the final version.

## Conflict of Interest Statement

The authors declare that the research was conducted in the absence of any commercial or financial relationships that could be construed as a potential conflict of interest.
